# Elevated Nrf-2 responses are insufficient to mitigate protein carbonylation in hepatospecific PTEN deletion mice

**DOI:** 10.1371/journal.pone.0198139

**Published:** 2018-05-25

**Authors:** Dennis R. Petersen, Laura M. Saba, Volkan I. Sayin, Thales Papagiannakopoulos, Edward E. Schmidt, Gary F. Merrill, David J. Orlicky, Colin T. Shearn

**Affiliations:** 1 Department of Pharmaceutical Sciences, School of Pharmacy, University of Colorado Anschutz Medical Campus, Aurora, Colorado, United States of America; 2 Department of Pathology, New York University School of Medicine, New York, New York, United States of America; 3 Perlmutter Cancer Center, New York University School of Medicine, New York, New York, United States of America; 4 Department of Microbiology and Immunology, Montana State University, Bozeman, Montana, United States of America; 5 Department of Biochemistry and Biophysics, Oregon State University, Corvalis, Oregon, United States of America; 6 Department of Pathology, School of Medicine, University of Colorado Anschutz Medical Campus, Aurora, Colorado, United States of America; University College London, UNITED KINGDOM

## Abstract

**Objective:**

In the liver, a contributing factor in the pathogenesis of non-alcoholic fatty liver disease (NASH) is oxidative stress, which leads to the accumulation of highly reactive electrophilic α/β unsaturated aldehydes. The objective of this study was to determine the impact of NASH on protein carbonylation and antioxidant responses in a murine model.

**Methods:**

Liver-specific phosphatase and tensin homolog (PTEN)-deletion mice (PTEN^LKO^) or control littermates were fed a standard chow diet for 45–55 weeks followed by analysis for liver injury, oxidative stress and inflammation.

**Results:**

Histology and Picrosirius red-staining of collagen deposition within the extracellular matrix revealed extensive steatosis and fibrosis in the PTEN^LKO^ mice but no steatosis or fibrosis in controls. Increased steatosis and fibrosis corresponded with significant increases in inflammation. PTEN-deficient livers showed significantly increased cell-specific oxidative damage, as detected by 4-hydroxy-2-nonenal (4-HNE) and acrolein staining. Elevated staining correlated with an increase in nuclear DNA repair foci (γH2A.X) and cellular proliferation index (Ki67) within zones 1 and 3, indicating oxidative damage was zonally restricted and was associated with increased DNA damage and cell proliferation. Immunoblots showed that total levels of antioxidant response proteins induced by nuclear factor erythroid-2-like-2 (Nrf2), including GSTμ, GSTπ and CBR1/3, but not HO-1, were elevated in PTEN^LKO^ as compared to controls, and IHC showed this response also occurred only in zones 1 and 3. Furthermore, an analysis of autophagy markers revealed significant elevation of p62 and LC3II expression. Mass spectrometric (MS) analysis identified significantly more carbonylated proteins in whole cell extracts prepared from PTEN^LKO^ mice (966) as compared to controls (809). Pathway analyses of identified proteins did not uncover specific pathways that were preferentially carbonylated in PTEN^LKO^ livers but, did reveal specific strongly increased carbonylation of thioredoxin reductase and of glutathione-*S*-transferases (GST) M6, O1, and O2.

**Conclusions:**

Results show that disruption of PTEN resulted in steatohepatitis, fibrosis and caused hepatic induction of the Nrf2-dependent antioxidant system at least in part due to elevation of p62. This response was both cell-type and zone specific. However, these responses were insufficient to mitigate the accumulation of products of lipid peroxidation.

## Background

In the United States, nonalcoholic steatohepatitis (NASH) resulting from unmitigated progression of non-alcoholic fatty liver disease (NAFLD) is rapidly becoming the major indicator for liver transplantation[1]. Increased lipid accumulation, inflammation and elevation of oxidative stress are hallmarks of NASH[2–4]. Pathologically, hepatic fat accumulation (steatosis) is frequently regarded as the initial insult during NAFLD and is hypothesized to be the prerequisite for progression to the inflammatory disease, steatohepatitis. Following steatosis, additional cellular processes including mitochondrial injury, oxidative stress and proinflammatory cytokines are all contributing factors in the progression of NAFLD to NASH[5].

The formation of reactive oxygen species (ROS) during chronic inflammation is central to the progression of chronic liver diseases[6]. While ROS are important in signal transduction, cellular physiology and critical metabolic pathways, a high concentration of ROS can result in macromolecular damage, apoptosis and necrosis[7]. Particularly damaging, ROS can initiate a free radical chain-reaction in unsaturated fatty acids, thereby generating toxic electrophilic α/β unsaturated aldehydes, a process called lipid peroxidation. The best characterized of these carbonyl-derivatives are 4-hydroxy-2-nonenal (4-HNE), malondialdehyde (MDA) and acrolein[8]. Following their formation, these highly reactive lipid aldehydes covalently link to nucleophilic Lys, Cys and His residues on proteins, exerting pathophysiological inhibitory effects. Levels of lipid peroxidation and post-translational modification of proteins by reactive lipid aldehydes (carbonylation) are reliable markers of oxidative stress[8–10].

The phosphatase and tensin homolog deleted on chromosome 10 (PTEN)/AKT pathway regulates lipogenesis in the liver[11]. PTEN itself is a dual specificity phosphatase possessing both lipid- and protein-phosphatase activity, and is a member of the protein tyrosine phosphatase family of phosphatases[12, 13]. Complete activation of AKT requires association of its pleckstrin homology domain with phosphatidylinositol (3,4,5) trisphosphate on cellular membranes. PTEN negatively regulates Akt activation through its ability to dephosphorylate the 3-position phosphate from phosphatidylinositol (3,4,5) trisphosphate to produce phosphatidylinositol (4,5) bisphosphate. Partial inactivation of PTEN leads to sustained AKT activation, increased mitochondrial respiration, and increased levels of reactive oxidative species (ROS) in mouse models[14].

In murine models, liver specific deletion of PTEN (PTEN^LKO^) is an established model to examine the effects of a NASH-like condition[15–17]. In validation of this model, human NASH shows decreased expression of PTEN as compared to normal human liver[16]. PTEN^LKO^ mice exhibit insulin hypersensitivity, hepatomegaly, biliary proliferation, elevated hepatic triglycerides, and constitutive de novo lipogenesis. Interestingly, a decrease in overall body fat occurs in contrast to a dramatic increase in hepatocellular steatosis[15, 17–20], suggesting that intermediary metabolism is severely impacted and energetic resources are being sequestered in the liver. As these mice age, they progress into steatohepatitis and fibrosis, and some animals spontaneously develop adenomas or hepatocellular carcinomas[17, 18].

We recently determined that consumption of a diet rich in polyunsaturated fatty acids for six-weeks significantly elevated hepatic oxidative stress in PTEN^LKO^ mice, which was in part due to increased mitochondrial respiration[21]. As important, in end-stage human NASH samples, protein carbonylation is significantly elevated[22] and the distribution of oxidatively damaged proteins was both cell type and zonally restricted. Specifically, staining of 4-HNE was elevated panlobularly, with the greatest increase in immunostaining occurring in areas adjacent to fibrotic tissue. In contrast, acrolein staining was increased in cholangiocytes as well as within hepatocytes surrounding fibrotic tissue with a mild increase throughout the rest of the lobule. In a recent study using the methionine and choline-deficient model of NASH, which is associated with elevated 4-HNE staining, the levels of both lipid peroxides and hepatocellular injury was significantly decreased by activation of the cytoprotective Nrf2-dependent antioxidant response pathway[23]. Importantly, PTEN^LKO^ mice also develop NASH, yet they exhibit enhanced activation of Nrf2-signaling[24]. In the present study, the effects of long term liver-specific PTEN deletion was examined in the context of oxidative stress responses and protein carbonylation. We show that protein carbonylation is elevated in PTEN^LKO^ livers as compared to controls, but that there are significant differences in the location of reactive aldehyde generation and protein carbonyl accumulation. We furthermore find that in the PTEN^LKO^ model, activation of Nrf2 is restricted with hepatoprotective responses both protein specific as well as hepatozonal-specific to areas of reactive aldehyde generation accumulation.

## Materials and methods

### Animal model

Hepatocyte-specific homozygous disruption of the conditional-null PTEN^fl^ allele (PTEN^LKO^) was driven by Alb-Cre[17, 25]. As controls, littermates lacking Alb-Cre were used. All mice were on a C57Bl6/J background and were maintained as previously described[21]. Mice of both sexes were used and these were maintained on standard chow (Harlan Laboratories #2020X, Madison, WI) for 45–55 weeks. At harvest, mice were injected with sodium pentobarbital and blood was collected from the inferior vena cava. Plasma was separated through centrifugation at 5000 rpm for 5 min at 4°C and was assayed for alanine aminotransferase (ALT) activity (Sekisui Diagnostics, P.E.I., Canada). Whole livers were excised and weighed. Caudate and median lobes were collected, fixed in 10% neutral buffered formalin and embedded in paraffin for histological and immunohistochemical (IHC) analyses. The remaining portion of the liver was homogenized and subjected to differential centrifugation and subcellular fractionation as previously described[10]. All animal protocols were approved by the Institutional Animal Care and Use Committee of the University of Colorado and were performed in accordance with published National Institutes of Health guidelines.

### Histological evaluation

Formalin fixed slides were analyzed following hematoxylin and eosin (H&E) or Picro-Sirius-Red (PSR) staining. For assessing inflammation and carbonylation, the following primary antibodies were used for IHC: rabbit polyclonal 4-HNE, rabbit polyclonal acrolein (Cell Sciences, Newburyport, MA), rat polyclonal F4/80 (Biorad, Hercules, CA), rat polyclonal B220 (BD Biosciences San Jose, CA), rabbit polyclonal CD3 (DAKO/Agilent Santa Clara, CA), rabbit polyclonal heme oxygenase (HO-1, Enzo Life Sciences, Farmingdale, NY), rabbit polyclonal phospho H2A.X (Cell Signaling, MA), rabbit polyclonal NQO-1 (Sigma, St. Louis, MO), goat polyclonal GSTμ (ABCAM,), rabbit polyclonal GSTπ, (MBL International, Woburn, MA), goat polyclonal myeloperoxidase (MPO) (Millipore, Billerica, MA)[10], rabbit polyclonal Glutathione Peroxidase 1 (Gpx1) (ABCAM, Cambridge, MA) and rabbit polyclonal Glutamate-Cysteine Ligase Catalytic Subunit (GCLC, Novus Biologicals, Littleton, CO), rabbit polyclonal Cbr1/3 (this study). Histologic images were captured on an Olympus BX51 microscope equipped with a four-megapixel Macrofire digital camera (Optronics; Goleta, CA) using the PictureFrame Application 2.3 (Optronics). All images were cropped and assembled using Photoshop CS2 (Adobe Systems, Inc.; Mountain View, CA).

### Western blotting

Western blotting for Glutathione Peroxidase 1 (Gpx1) (ABCAM, Billerica, MA), GSTπ (MBL International, Woburn, MA), GSTμ (ABCAM, Cambridge, MA), Heme Oxygenase 1 (HO-1) Enzo Life Sciences, Farmingdale, NY, GCLC (Novus Biologicals, Littleton, CO), P62 (SQSTM1, Sequestosome 1) mouse monoclonal (ABCAM), LC3B (Novus Biologicals), anti-GAPDH (Millipore, Billerica, MA) was performed from 10 μg of liver extracts as previously described [10, 26–28]. Quantification of expression of each protein was performed using ImageJ (NIH) and normalized to overall GAPDH expression.

### Biotin hydrazide purification of carbonylated proteins

Liver extracts (LE) (500 μg) were prepared from age matched hepatic tissue obtained from control and PTEN^LKO^ mice (n = 6/genotype). Aldehyde-modified proteins from each extract were derivatized using biotin hydrazide (BH)(ThermoFisher/Pierce, Waltham, MA)(5 mM/2 hrs/room temperature/dark) followed by NaBH_4_ reduction (10 mM/100 mM NaOH 1hr/dark). BH-linked carbonylated proteins were incubated overnight using monoavidin columns (ThermoFisher/ Pierce). Columns were washed 5X in phosphate buffered saline (PBS) pH 7.4, 5X in PBS 0.5 M NaCl (pH 7.4) and 5X in PBS/2 M urea (pH 7.4) and eluted with 6X200 microliters of 0.2 M NH_4_OH. Elutions were pooled and dried down using a roto-evaporator (Thermo Scientific/Savant) and boiled in 6X SDS PAGE loading buffer (15 minutes). Samples were loaded on 10% SDS PAGE gels and run at 160V for 20 min. Gels were removed and stained with Coomassie blue overnight (Imperial Stain, ThermoFisher/Pierce) followed by destaining in ddH_2_O overnight. Bands containing carbonylated proteins were excised, destained, and digested with trypsin as previously described [29]. Following digestion, peptides were extracted, dried down, and resuspended in 0.1% formic acid in ddH_2_O.

### LC-MS/MS analysis

For LC-MS/MS analysis, 8 μl of each peptide mixture was loaded on a Bruker Amazon Speed LC-MS/MS and a Bruker Maxis IMPACT LC-MS/MS. The instrument was operated using data-dependent collision-induced dissociation (CID) MS/MS or electron transfer dissociation (ETD) with a threshold of 10,000 total ion current (TIC). Data analysis was performed using ProteinScape V3.1.2 (Bruker Daltonics Inc. Billerica, MA) and Mascot (v2.1.04, Matrixscience). First a global search for proteins was conducted with a Mascot cutoff score of 80 with the following variable modifications of carbamidomethly (C) and oxidized (M). After the initial search a second search was conducted for post-translational modification by reactive aldehydes using the masses as described previously. For this 2^nd^ iteration, peptide significance required a Mascot score higher than 26 and visual validation of spectra.

### Bioinformatic analysis of carbonylated proteins

Functional enrichment of carbonylated proteins for KEGG pathways [30, 31] and Gene Ontology Terms [32] was tested using a one-sided Fisher Exact Test in each experimental group (2 genotypes X 3 treatment conditions = 6 experimental groups). Differential enrichment between experimental groups was examined using a two-sided Fisher Exact test. For KEGG pathway enrichment, proteins were mapped to pathways using their Uniprot ID and databases were downloaded from the KEGG API (downloaded 7/14/2017). For Gene Ontology Terms, gene annotations were downloaded directly from the Gene Ontology website (http://www.geneontology.org; GOC Validation Date—08/28/2017) and Uniprot IDs were converted to gene symbols using the biomaRt package in R [33]. All analyses were executed in R (version 3.4.1).

### Statistical analysis

The data are presented as means ± SE. Comparisons between genotype was accomplished by one-way ANOVA, followed by Student Newman-Keuls *post hoc* analysis. Comparisons between two groups were accomplished using Student’s T-tests. Statistical significance was set at P<0.05. Prism 5 for Windows (GraphPad Software, San Diego, CA) was used to perform all statistical tests.

## Results

### Effects of PTEN^LKO^ on hepatocellular injury

We previously showed that the consumption of a diet high in polyunsaturated fatty acids significantly increased hepatocellular injury in PTEN^LKO^ mice[21, 34]. Furthermore, injury increases as PTEN^LKO^ mice age with the formation of hepatocellular carcinoma or adenomas as a frequent outcome[18, 35, 36]. To determine the biochemical pathology in older PTEN^LKO^ animals, serum alanine aminotransferase (ALT) and liver-to-body weights were determined. As shown in Part A in S1 Fig, serum ALT increased almost 8-fold in the PTEN^LKO^ group. Furthermore, both liver weight and liver to body weight were increased 3-4-fold in PTEN^LKO^ mice as compared to the control group (Part B and Part C in S1 Fig).

**Fig 1:**
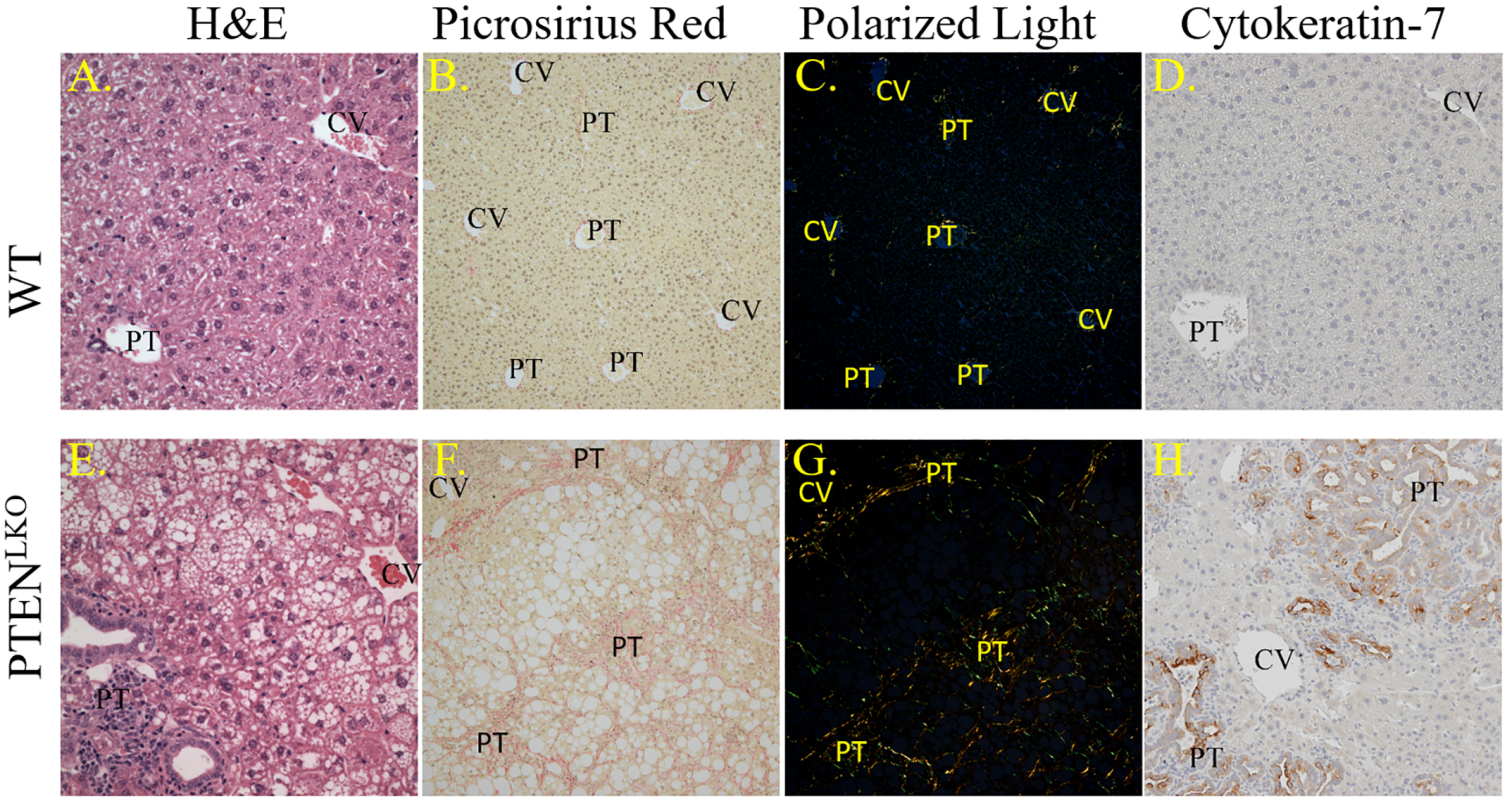
Basic pathology in PTEN^LKO^ livers. Panels A, E. Histology of livers from control or PTEN^LKO^ mice. Panels B, F. Bright-field images of PSR-staining. Panels C, G. Polarized light exposure of PSR-staining. Panels D, H. Cytokeratin 7 staining. Representative images are shown; n = at least 3 mice per genotype (CV, central vein, PT, portal triad).

The progression of NAFLD to NASH frequently leads to an increase in fibrosis. To examine the extent of fibrosis in the older PTEN^LKO^ mice, liver sections were analyzed histologically after staining with H&E or PSR. Neither steatosis nor fibrosis were present in the WT animals (Fig 1A and 1B). In the PTEN^LKO^ group, dramatic steatosis with fibrosis was observed, which was most predominant around the portal triads (PT; Fig 1E and 1F). Polarized light images[37] further revealed the dramatic increases in PSR intensity in PT regions (Fig 1C and 1G). Quantification of polarized images revealed that the PTEN^LKO^ possessed 5-fold higher PSR staining when compared to WT (S1 Table). The polarized light images also revealed predominantly a peri-portal localized fibrotic reaction with bridging fibrosis (portal area to portal area) in aged PTEN^LKO^ mice.

Previous studies using Cytokeratin-19-staining have shown that PTEN^LKO^ mice possess a strong ductular response as bile ducts proliferate in response to constitutive Akt activation [36]. To further explore the ductal response in aged PTEN^LKO^ mice, tissue sections were stained with another marker of the ductular reaction, cytokeratin-7 (CK7). CK7 staining was prominent in the expansive bile duct cholangiocytes or closely associated with these ducts indicating their hyperplasia in the PTEN^LKO^ group (Fig 1D and 1H), verifying that these livers had a strong ductal reaction. Quantification of CK7+ cells revealed an 8-fold increase in PTEN^LKO^ (S1 Table).

### Effects of PTEN^LKO^ on infiltration of inflammatory cells

**Fig 2:**
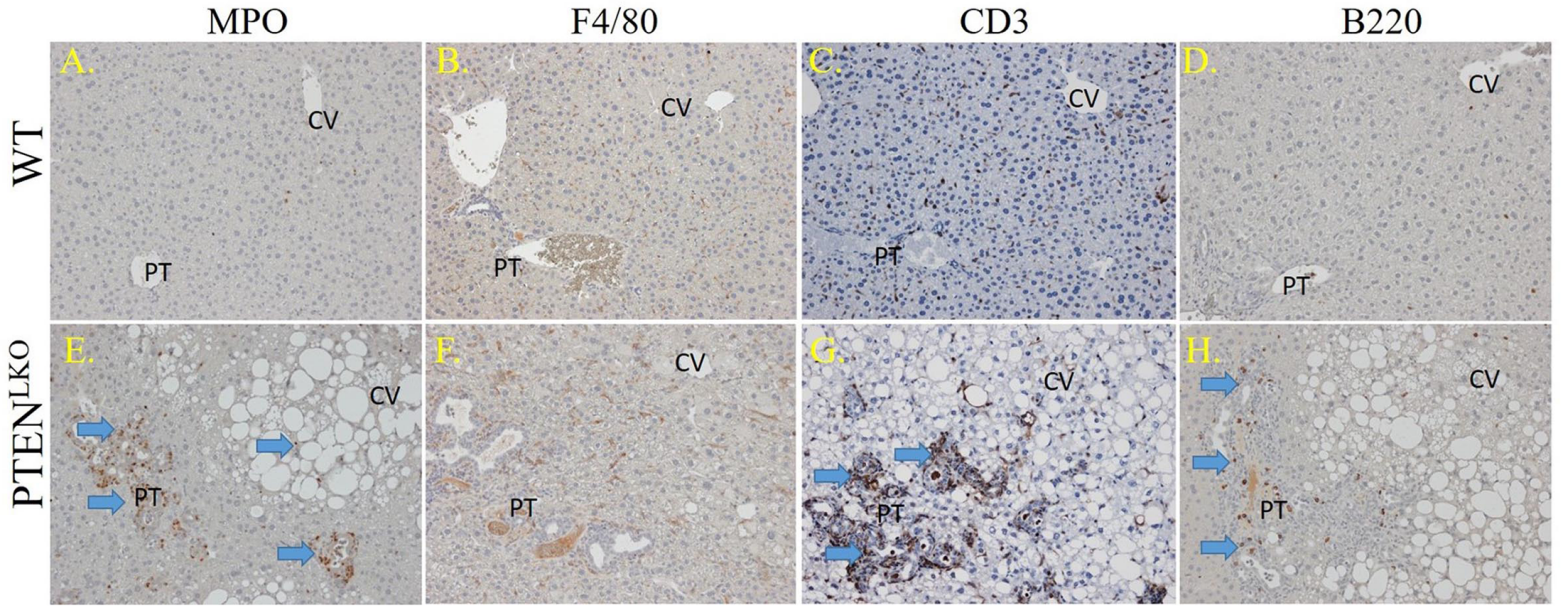
Inflammatory infiltrates in PTEN^LKO^ livers. Liver sections from control and PTEN^LKO^ mice were immunostained using antibodies directed against MPO (panels A, E); F4/80 (panels B, F); CD3 (panels C, G); or B220 (panels D, H). Representative images are shown; n = at least 3 mice per genotype. Abbreviations as in Fig 1.

Increased inflammation and the accumulation of reactive oxidative species (ROS) are integrally linked in NASH[38]. To assess the impact of increased steatosis and fibrosis on hepatocellular inflammation, tissue sections were probed for myeloperoxidase (MPO, a marker of activated neutrophils), F4/80 (a marker of infiltrating macrophages and resident Kupffer cells), B220 (a B cell marker) and CD3 (a lymphocyte marker). Few MPO-positive neutrophils were present in control livers, whereas in PTEN^LKO^ livers, a dramatic increase in MPO positive cells occurred both in areas undergoing ductular reactions as well as within strongly steatotic tissue (Fig 2A & 2E, S1 Table). A large increase in F4/80-positive cells was also evident in PTEN^LKO^ as compared to control liver (Fig 2B & 2F, S1 Table). Collectively, these data reveal a strong inflammatory response in PTEN^LKO^ livers. Recent reports have indicated that the adaptive immune response also contributes to oxidative stress in fatty NASH[3], so lymphocytes were also examined in PTEN^LKO^ and control livers. Although in control livers, B220+ plasma/B-lymphocytes and CD3+ lymphocytes were rare and randomly dispersed, in PTEN^LKO^ livers, B220+ lymphocytes and CD3+ lymphocytes were abundant in regions of ductular responses and throughout areas of increased steatosis (Fig 2C, 2D, 2G & 2H arrows), verifying that there are both innate and adaptive immune responses in areas around proliferating cholangiocytes in PTEN^LKO^ livers. Interestingly, quantification of CD3+ cells revealed a significant accumulation within the periportal region (S1 Table).

### Impact of PTEN^LKO^ on protein carbonylation

**Fig 3:**
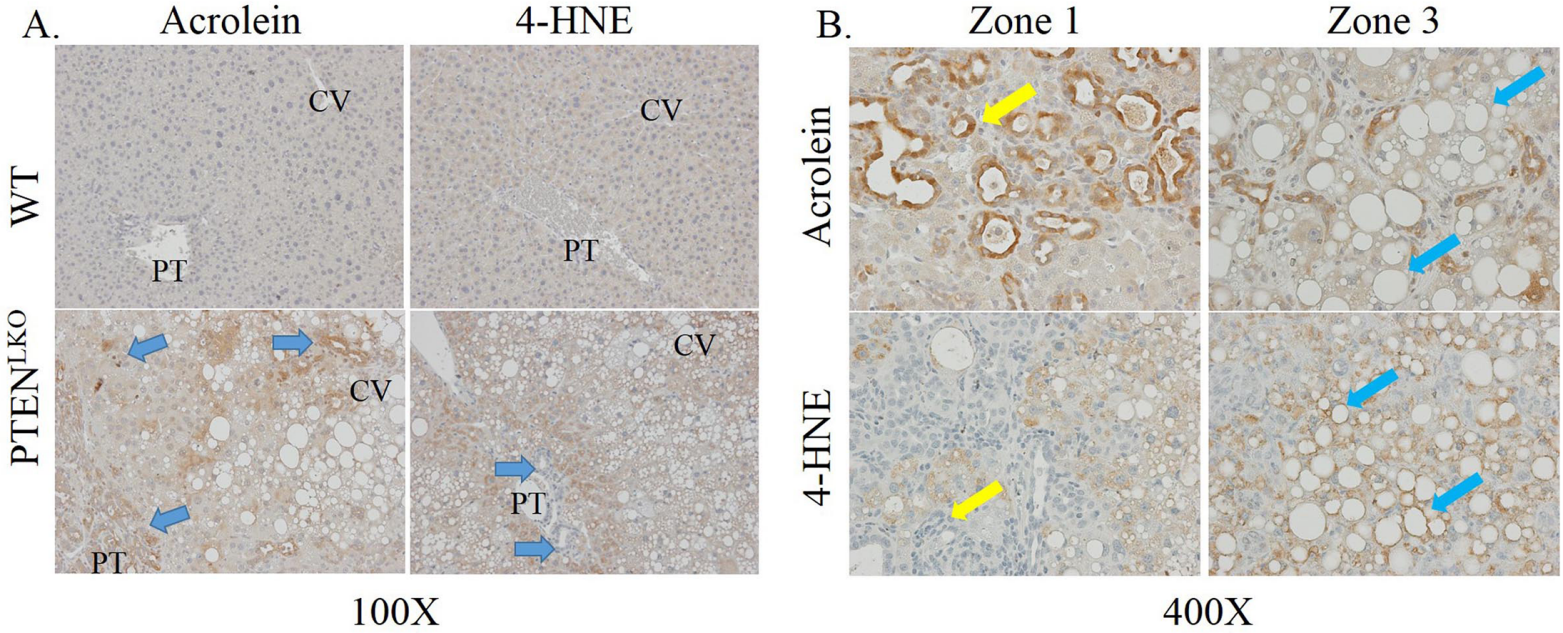
Impact of PTEN^LKO^ on protein carbonylation. Tissue sections isolated from control and PTEN^LKO^ mice were immunostained using antibodies directed against 4-HNE or acrolein, as indicated. A. 100X magnification, B. 400X magnification of PTEN^LKO^ livers. Representative images are shown; n = at least 3 mice per genotype. Abbreviations as in Fig 1.

Protein carbonylation is proposed to play a key role in the progression of NASH[5]. Tissue sections isolated from PTEN^LKO^ and control mice were probed for protein modification by the reactive aldehydes acrolein and 4-HNE using IHC. In healthy control liver, both acrolein and 4-HNE showed very mild or background staining, which was evenly dispersed across the lobule (Fig 3A). Tissue sections isolated from PTEN^LKO^ revealed dramatically increased 4-HNE staining in centrilobular and peri-portal hepatocytes but not in cholangiocytes (yellow arrows) or in zone 2. This was in contrast to acrolein-staining, which was notably increased in cholangiocytes, inflammatory cells, and in zones 1 and 3 (blue arrows). Higher magnification images more clearly showed that acrolein staining was present within hepatocytes but not around steatotic vesicles, whereas 4-HNE specifically stains the outside of vesicles (Fig 3B, compare blue and yellow arrows). Quantification of staining revealed a significant increase in both 4-HNE and acrolein staining (S1 Table).

### Impact of PTEN^LKO^ on cellular oxidative damage and proliferation

**Fig 4:**
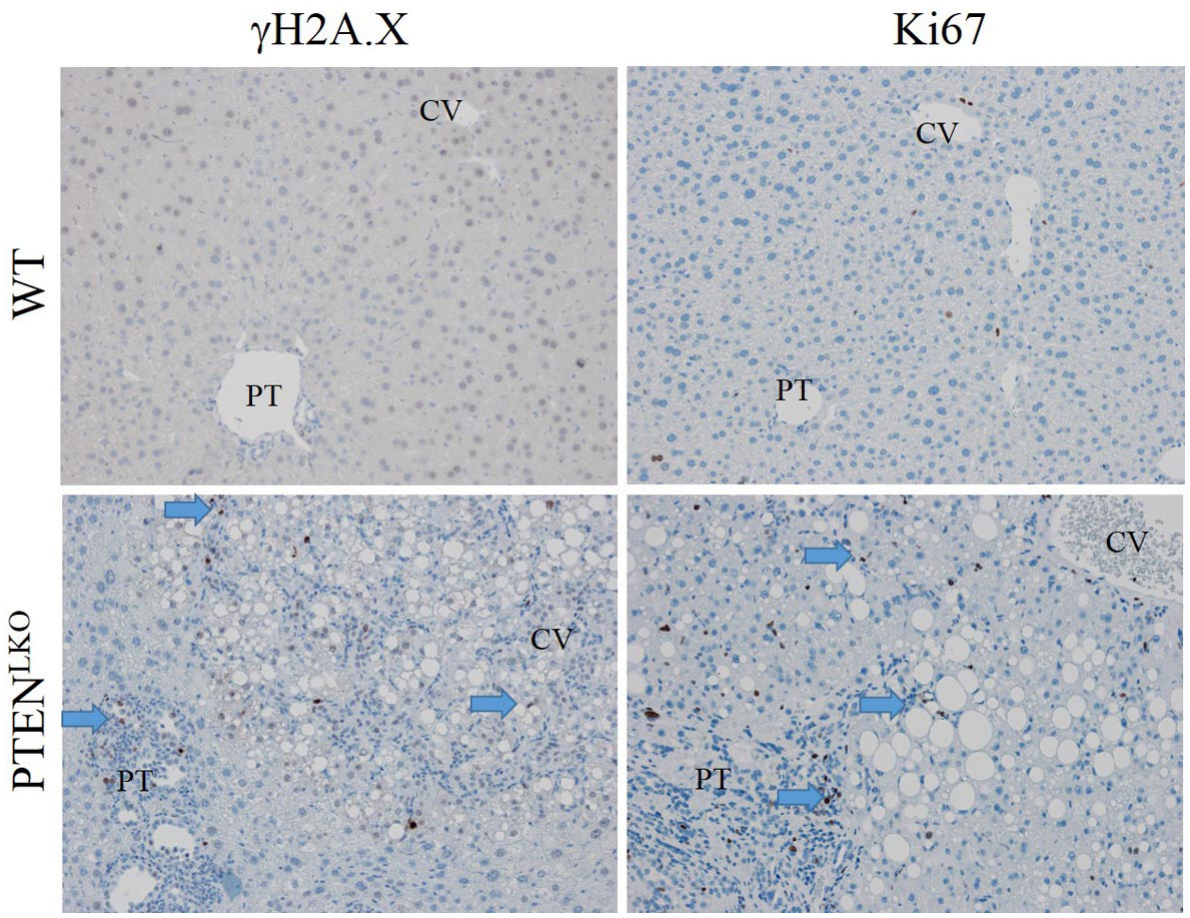
Impact of PTEN^LKO^ on DNA double-strand break repair and proliferation. Tissue sections isolated from control and PTEN^LKO^ mice were immunostained using antibodies directed against γH2A.X and Ki67. N = at least 3 mice per genotype. Representative images are shown; n = at least 3 mice per genotype. Abbreviations as in Fig 1.

In PTEN^LKO^ mouse livers, there was strong acrolein staining both in the cytosol and the nuclei of affected cholangiocytes. In addition to proteins, reactive aldehydes including 4HNE, MDA, and acrolein can also modify DNA resulting in DNA damage and double stranded DNA breaks. To assess whether this was occurring in the PTEN^LKO^ mouse livers, we stained for the double stranded DNA break-marker histone γH2AX. H2A.X-staining was prominent within both a subset of periportal cholangiocytes (arrows) as well as in hepatocytes within zones 1 and 3 in PTEN^LKO^ mice, but nearly absent in control livers (Fig 4, upper panels, S1 Table), and in the case of the cholangiocytes, it correlated to the hyperproliferative ductal response seen in PTEN^LKO^ mice (see above). Furthermore, staining was evident in both the periportal region as well as areas of severe central venous steatosis but not in zone 2. Oxidative stress is also associated with increased oval cell proliferation[39]. To determine if cells that display DNA damage and protein-carbonylation were also undergoing proliferation, tissue sections were stained for ki67. As shown in Fig 4 lower panels, S1 Table, increased Ki67+ cells were prominent in both the periportal and centrilobular regions in PTEN^LKO^ mice, although not necessarily within the same cells.

### Lobular zone-specific impact of PTEN^LKO^ on the Nrf2 anti-oxidant response

**Fig 5:**
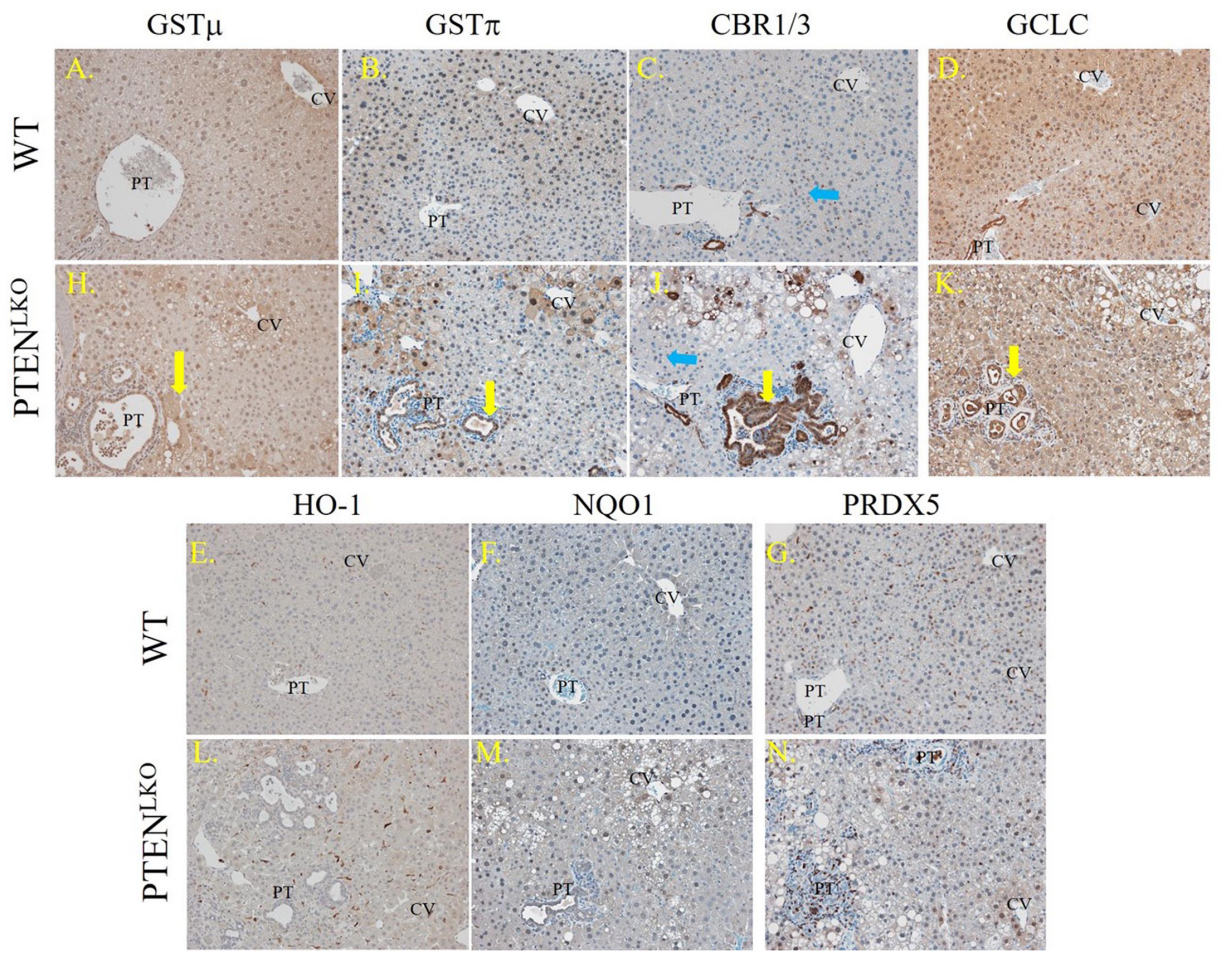
Impact of PTEN^LKO^ on zonal expression of antioxidant response enzymes. Liver sections from control and PTEN^LKO^ mice were immunostained using polyclonal antibodies directed against the indicated markers: Representative images are shown; n = at least 3 mice per genotype. Abbreviations as in Fig 1.

We hypothesized that the observed increase in cellular oxidative damage would result in upregulation of oxidative detoxification enzymes. The impact of elevated carbonylation on zone-specific levels of GSTμ, GSTπ, CBR1/3 and the Glutamate-Cysteine Ligase Catalytic Subunit (GCLC) was assessed using IHC (Fig 5A–5D and 5H–5K). Control livers showed mild GSTμ staining in areas surrounding the central vein and in some nuclei, whereas in PTEN^LKO^ livers, GSTμ staining was dramatically increased in hepatocytes within areas of active steatosis (centri-lobular, zone 3), as well as in many cholangiocytes within the bile ducts undergoing a ductular response (yellow arrow). Examining GSTπ, in the control mice, cholangiocytes exhibited moderate GSTπ staining whereas weak GSTπ staining was evident throughout the centri-lobular hepatocytes. In comparison, in PTEN^LKO^ mice, GSTπ staining was elevated in cholangiocytes found in the hypertrophied bile ducts (yellow arrow), periportal hepatocytes as well as centri-lobular hepatocytes. In control mice, CBR1/3 staining was evident in cholangiocytes within the portal tract as well as within Kupffer cells (blue arrow) throughout the lobule. In comparison, in PTEN^LKO^ mice, a significant elevation of CBR1/3 staining was noted in cholangiocytes in the hypertrophied bile ducts (yellow arrow) as well as in periportal and central venous hepatocytes. Examining GCLC localization, in WT mice, GCLC exhibited mild centrilobular staining and was also present in resident Kupffer cells, lymphocytes and cholangiocytes. In PTEN^LKO^ mice, GCLC staining was elevated within proliferating cholangiocytes and within centri-lobular hepatocytes. In addition to cytosolic staining, all four proteins (GSTμ, GSTπ CBR1/3 and GCLC) exhibited significant nuclear localization.

In a previous report using PTEN^LKO^ mice, increased nuclear localization and activation of Nrf2 was observed[24]. Many glutathione-S-transferases, in particular in the A & M families, are downstream targets of Nrf2[40]. Loss of PTEN induces an Nrf2 response, which stimulates cellular proliferation by a KEAP1-independent mechanism[41]. To explore the impact of PTEN^LKO^ on Nrf2 anti-oxidant responses, tissue sections were stained for additional Nrf2-induced proteins, including HO-1, NQO-1, as well as the unrelated anti-oxidant protein Prdx5 (Fig 5E–5G and 5L–5N) using polyclonal antibodies[42, 43]. Examining HO-1, hepatocytes exhibited weak HO-1 staining that was barely perceptible. Instead, HO-1 staining was present panlobularly within Kupffer cells that were predominantly within the sinusoids. Interestingly, HO-1 staining was not increased in PTEN^LKO^ mice. In WT, staining of NQO-1 was mildly evident in hepatocytes surrounding the central vein and in cholangiocytes. Deletion of PTEN resulted in an increase in NQO1 staining within nuclei of hepatocytes throughout the lobule. Furthermore, cytosolic NQO1 staining also increased within proliferating cholangiocytes and in centrilobular hepatocytes. Expression of Prdx5 has not previously been reported in mice. Tissue sections obtained from control livers demonstrated Prdx5 expression within centrilobular hepatocytes, resident Kupffer cells and cholangiocytes. In PTEN^LKO^ livers, Prdx5 expression was elevated in both the centrilobular and periportal regions.

### Impact of PTEN^LKO^ on expression of anti-oxidant responses

**Fig 6:**
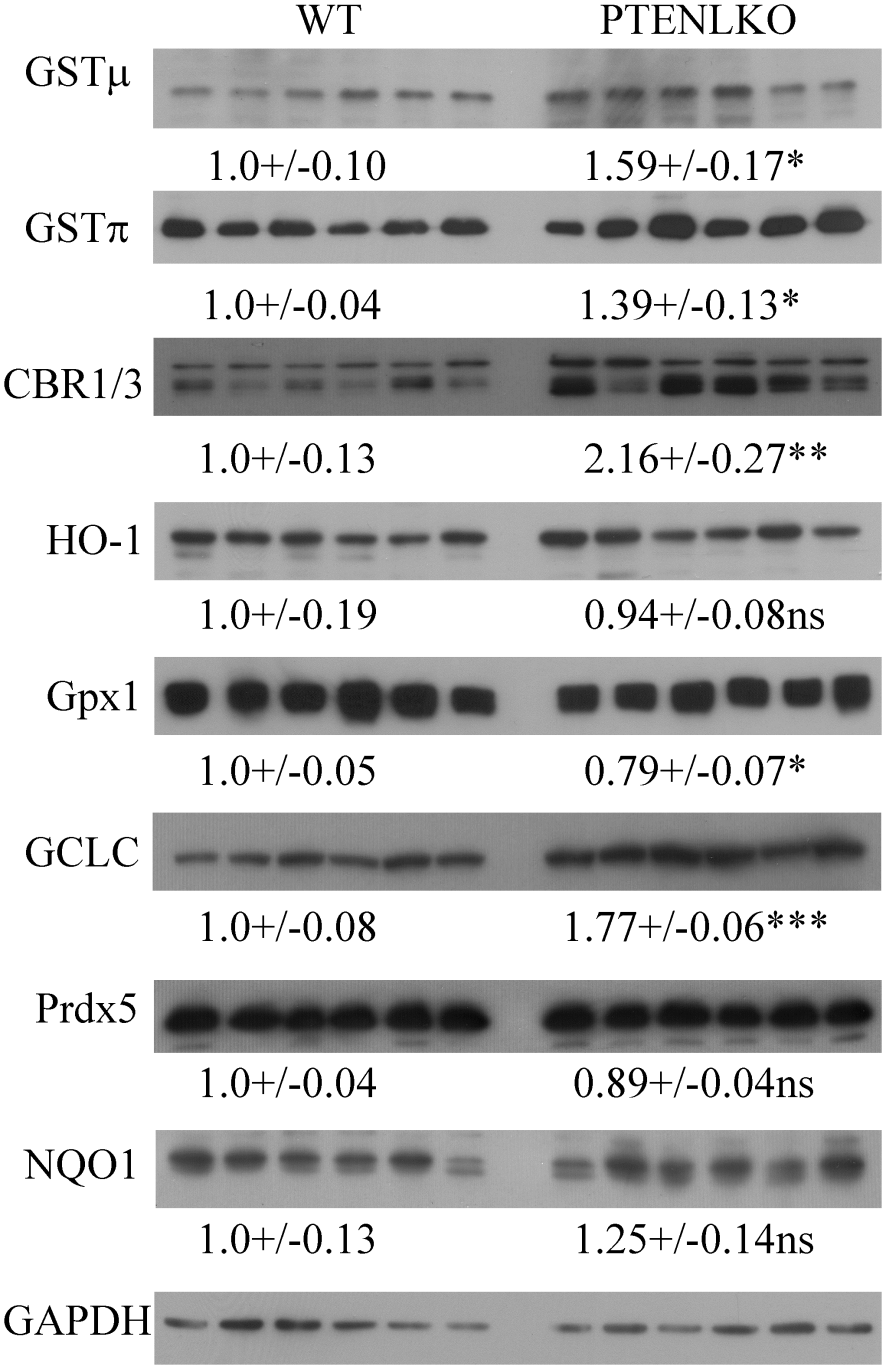
Impact of PTEN^LKO^ on expression of antioxidant responses. Western immunoblotting analysis of GCLC, GSTμ, GSTπ, CBR1/3, HO-1, Prdx5 and Gpx1 in liver lysates prepared from control and PTEN^LKO^ mice. All exposures were normalized using GAPDH expression. Data are means +/- SEM, n = 6 per genotype.

From histology, increased staining of CBR1/3, GCLC, GSTμ and GSTπ was evident in PTEN^LKO^ livers. To further support histological data, the hepatic expression of CBR1/3, GCLC, GSTμ, GSTπ, HO-1, Prdx5 and Gpx1 was assessed using liver extracts and Western blotting. In PTEN^LKO^ mice, a significant increase in CBR1/3, GCLC, GSTμ, GSTπ was evident, but there was no change in HO-1 or Prdx5 expression and Gpx1 was suppressed by 20% (Fig 6).

### Impact of PTEN^LKO^ on expression of autophagic responses

**Fig 7:**
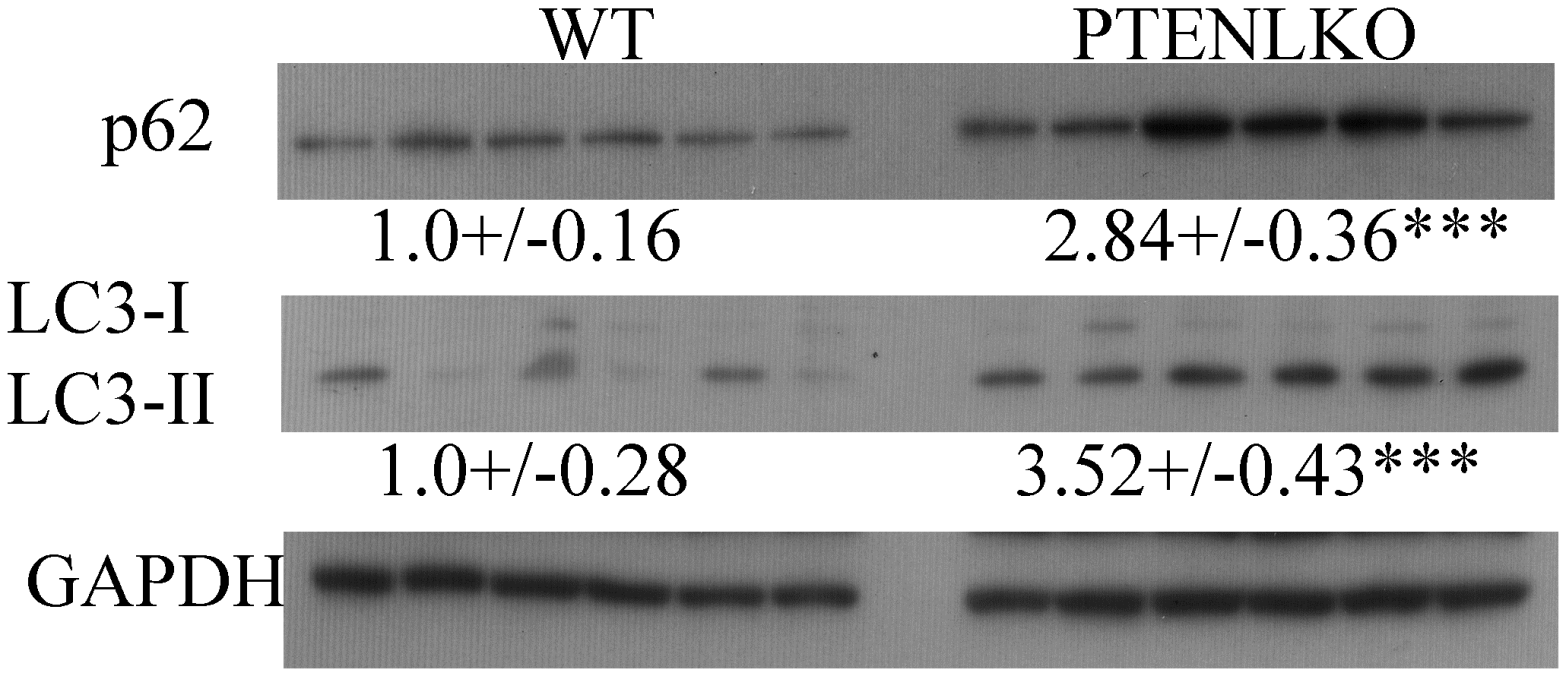
Impact of PTEN^LKO^ on expression of markers of autophagy. Western immunoblotting analysis of p62, LC3I and LC3II in liver lysates prepared from control and PTEN^LKO^ mice. All exposures were normalized using GAPDH expression. Data are means +/- SEM, n = 6 per genotype.

Histology and Western blotting indicates upregulation of Nrf2-specific antioxidant responses. Interestingly, mice that are defective in autophagy possess hepatomegaly and elevated Nrf2 activation [44, 45]. A critical characteristic of autophagy is an increase in lipid degradation and promotion of fibrosis. To determine the impact of PTEN^LKO^ on autophagic processes, liver extracts were examined for p62, LC3I and LC3II expression using Western blotting (Fig 7). In PTEN^LKO^ mice a significant increase in p62 and LC3-2 expression was evident.

### Proteomic analysis of carbonylated proteins in PTEN^LKO^ mice

We recently reported the use of BH-derivatization followed by global LC-MS/MS analysis to identify carbonylated proteins in subcellular fractions isolated from livers of pair-fed and ethanol-fed mice and human NASH patients[22, 46]. In the present study, BH-derivatization was performed on extracts prepared from PTEN^LKO^ versus control livers. Following affinity purification, carbonylated proteins were digested with trypsin, and peptides were analyzed by LC-MS/MS. Collectively, **1107** carbonylated proteins were identified in the samples (S2 Table). Of these, **92.3**% had been previously identified in other murine models of chronic hepatocellular inflammation [29, 47]. Interestingly, **78** proteins presented in S3 Table, had not been previously identified as carbonylated in murine models of alcoholic liver disease using a similar mass spectrometric approach[29, 46].

**Fig 8:**
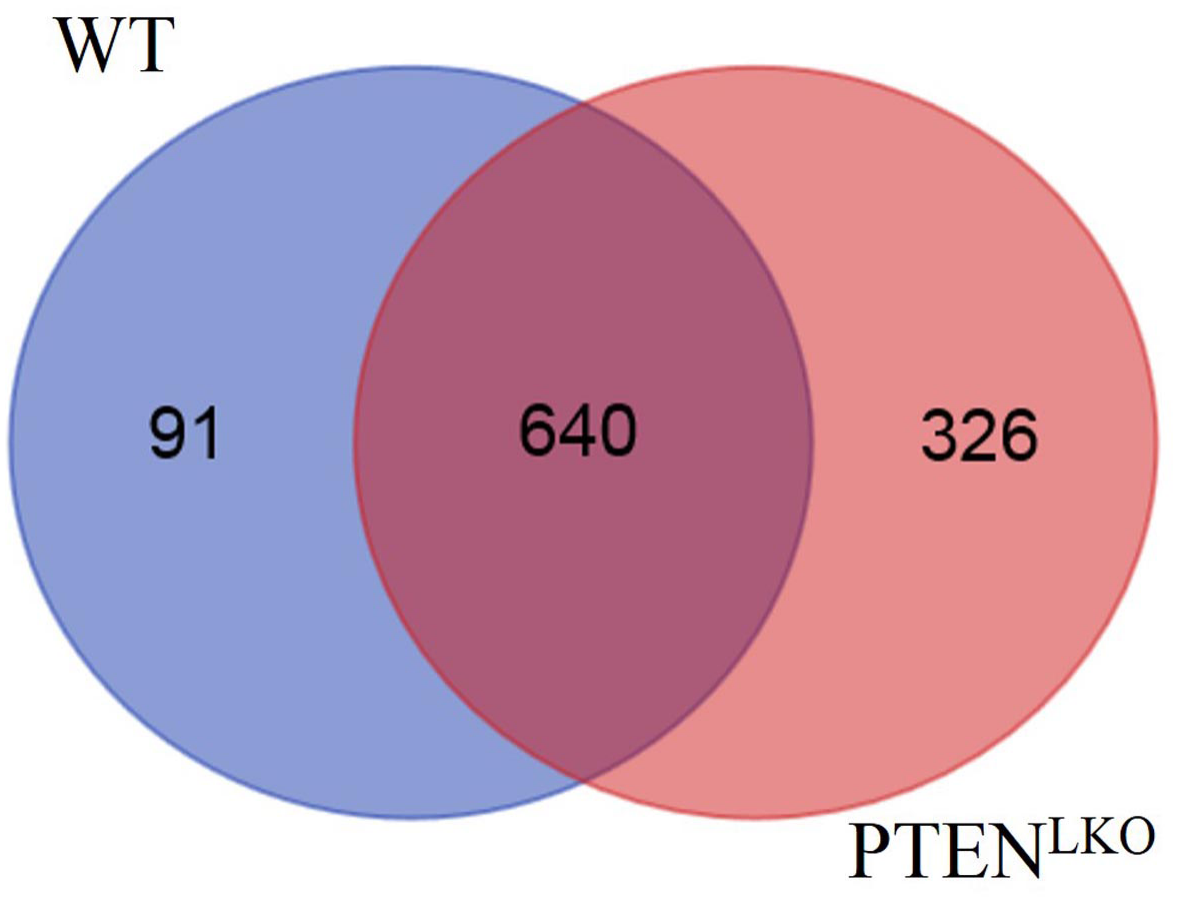
VENN analysis of carbonylated proteins in control and PTEN^LKO^ livers.

VENN analysis of the data revealed that 668 carbonylated proteins were common to both control and PTEN^LKO^ livers, 141 carbonylated proteins were found only in the control livers, and 298 carbonylated proteins were found only in the PTEN^LKO^ livers (Fig 8, S4 Table). A complete list of the proteins unique to PTEN^LKO^ mice is presented in S5 Table. Interestingly, antioxidant proteins, including thioredoxin reductase, GSTμ6, GSTΩ1 and GSTθ2, were only carbonylated in PTEN^LKO^ livers. To identify cellular pathways preferentially impacted by carbonylation in PTEN^LKO^, these datasets were subjected to differential enrichment analysis using Gene Ontology and KEGG pathways [30, 48, 49]. Surprisingly, these analyses did not reveal significant differences in any specific pathways, but rather, suggested that the effect of PTEN^LKO^ deletion on protein carbonylation was more broadly distributed.

## Discussion

Our data demonstrate increased lipid peroxidation in the periportal (zone 1) and centrilobular (zone 3) regions of PTEN^LKO^ livers, but not in the intervening zone 2 hepatocytes. This increase in lipid peroxidation correlated with both a co-localized increase in mitochondrial respiration and with increased infiltration of inflammatory cells; both likely contributing to a localized increase in ROS. Increased ROS, combined with the increase in lipid synthesis previously reported in PTEN^LKO^, provide an environment that would favor formation of lipid peroxides. Interestingly, acrolein staining was significantly increased in proliferating cholangiocytes, yet these cells did not accumulate 4-HNE. Also, in zone 3, 4-HNE staining is adjacent to vesicular membranes where we detected no acrolein. This indicates that the source of short chain aldehydes (SCA) such as acrolein is different than that of longer aldehydes, such as 4-HNE, which has been hypothesized to originate from linoleic acid within the membranes[50]. Moreover, staining for both aldehydes contrasts with reported lipid peroxidation in early stages of NASH, which is primarily evident in the centrilobular (zone 3) region[51]. More work will be needed to resolve the causes of this apparent discrepancy.

Recent evidence has provided a direct link between PTEN and activation of Nrf2 cellular responses. Suppression of PTEN causes increased levels of Nrf2 and its target gene, NQO-1, in cells following exposure to the classic Nrf2-inducer, tert-butylhydroquinone (tBHQ)[52]. In cancer, sustained activation of the PTEN target AKT increased Nrf2 nuclear localization[53]. In PTEN+/- mice, Nrf2 expression is increased, thereby upregulating anti-oxidant responses and providing a growth advantage to proliferating cholangiocytes[24]. In chow-fed PTEN^LKO^ mice, basal hepatic concentrations of GSH are increased, consistent with these livers having activation of GSH synthesis by the Nrf2 target GCLC[21]. Yet, by its ability to reduce products of oxidation, elevated GSH can also act as a negative regulator of Nrf2[54]. This is not evident in PTEN^LKO^.

The role of the PTEN/AKT pathway in cellular autophagic processes has been well described[55]. PTEN regulates cellular concentrations of phosphatidylinositol 3,4,5P_3_ initiating the formation of the autophagosome. In cell culture, initiation of DNA damage and accumulation of ROS activates PTEN thereby promoting autophagy[56]. In contrast, activated AKT will phosphorylate mammalian target of rapamycin (mTOR) suppressing autophagy. Therefore, we anticipated that autophagy would be repressed in PTEN^LKO^ mice. Surprisingly, as evidenced by increased abundance of LC3II, our data support upregulation of both oxidative stress and autophagy occurs in PTEN^LKO^ despite persistent AKT activation. This finding is contrasted by an increase in p62 expression indicating that there may be dysregulation. Recent evidence has demonstrated that by the ability of p62 to interact with the Nrf2 negative regulator Keap1, Nrf2 activation can be regulated by autophagic processes[45]. This provides a direct link between cellular oxidative stress and autophagy[57]. Importantly, mice that are deficient in autophagy due to hepatospecific deletion of both Atg7 and Atg5 also exhibit persistent Nrf2 activation, elevated triglycerides and hepatomegaly similar to PTEN^LKO^[44]. From our data, this alternative mechanism of Nrf2 activation is also occurring in PTEN^LKO^ further supporting the link between oxidative stress, Nrf2 activation and autophagy.

Previous reports showed that GSTπ, GSTμ and CBR1/3 each participate in lipid peroxide detoxification[58–62]. In humans, chronic EtOH consumption increases GSTμ expression whereas GSTμ null alleles correlate with increased risk for developing NAFLD[63, 64]. We find that all of these proteins are increased in both proliferating cholangiocytes and in centrilobular hepatocytes of PTEN^LKO^ livers, suggesting they may participate in detoxification in both cell types. Concurrently, we find GCLC expression is also increased in the same cells. This is in agreement with our previously published report that demonstrated elevated concentrations of both GSH and GSSG in liver tissue isolated from PTEN^LKO^ mice[21]. This is also the location where increased staining of 4-HNE and acrolein was measured. With the strong accumulation of acrolein in cholangiocytes, acrolein staining is noticeably different and colocalizes with GSTμ, GSTπ and CBR1/3. Surprisingly the increased levels of these cytoprotective enzymes, in concert with the previously reported increased levels of GSH, is still not adequate to fully mitigate the accumulation of reactive aldehyde species, including 4-HNE, MDA, and acrolein, in aged PTEN^LKO^ mice.

Heme oxygenase 1 (HO-1) is an enzyme that catalyzes the breakdown of heme to biliverdin, thereby releasing carbon monoxide[65, 66]. HO-1 expression is induced during conditions of increased oxidative stress as well as in response to cytokines[67]. As both bilirubin and CO can have antioxidant activity, HO-1 is considered a cytoprotective antioxidant enzyme[68]. In human patients with NASH as compared to either normal patients or patients with simple steatosis, HO-1 expression and lipid peroxidation levels are strongly elevated, GSH levels are diminished [69]. The authors concluded that HO-1 induction is an adaptive response against oxidative damage that results from lipid peroxidation[69]. In murine NASH models, HO-1 levels can be either elevated or suppressed, depending on the model[70–72]. For example, in 32 week high fat fed PTEN^+/-^ livers, HO-1 expression is increased and correlates with elevated Hsp70 expression and oxidative stress[14]. By contrast, in PTEN^LKO^ livers we do not see elevated HO-1 expression. Indeed, our data show that HO-1 is primarily expressed in Kupffer cells, not in hepatocytes (Fig 5). Consistent with our findings, liver-specific Keap1 deletion, which leads to chronic Nrf2 induction, causes elevated GCLC and GSTA4 expression in the liver, but does not affect HO-1 levels[73]. Thus, in PTEN^LKO^ livers, HO-1 does not appear to participate in the protective responses to lipid peroxidation.

Finally, our data show that the increases in lipid peroxidation in the PTEN^LKO^ livers was associated with dramatic increases in double stranded DNA damage in both hepatocytes and cholangiocytes, as evidenced by γH2A.X staining (Fig 4). This suggested that both cell types in these livers experienced increased nuclear oxidative damage and, indeed, our data also showed increased levels of nuclear acrolein in cholangiocytes. Modifications of DNA by lipid peroxides is known to cause base pair substitutions that can lead to misrepair[74, 75]. Interestingly, accumulation of misrepaired DNA lesions is a prerequisite of carcinogenesis[76, 77], so our data provide a compelling mechanistic explanation for the high incidence of spontaneous liver adenomas and hepatocellular carcinomas observed in the aged PTEN^LKO^ mice.

In conclusion, results herein provide evidence that in PTEN^LKO^, there is increased activation of proteins capable of mitigating the accumulation of protein carbonyls, but that this increase is not sufficient to fully abrogate the accumulation of oxidative damage to proteins or to DNA. Interestingly, the different products of lipid peroxidation that we measured in the PTEN^LKO^ livers occurred in different hepatocellular zones and well as different cell types within each region, suggesting cells of each type or each zonal location had distinct innate abilities to defend themselves against lipid peroxide-induced damage.

## Supporting information

S1 Fig Liver injury and liver weight and liver to body weight ratio in PTEN^LKO^ mice. A. Alanine aminotransferase. B. Liver Weight. C. Liver to Body weight ratio. Data are means +/- SEM. N = 8 PTEN^LKO^, 9 controls.
(TIF)

S1 Table Quantification of pathology. For MPO, B220, Ki67 and pH2A.x, positively stained cells were counted per 40X field. For F4/80 and CK7, positively stained cells were counted per 100X field. For CD3+ quantification, 6–10 40x images from each mouse were then imported into the program "SlideBook" (3I, Denver, Colorado). Each image was segmented and then the total number of pixels of the PT area and the number of pixels that are positive were identified. For PSR quantification, a minimum of 4 40x images of Picro-Sirius stained liver tissues using incident polarized light were imported "SlideBook" (3I, Denver, Colorado). Each image was segmented by defining the image/nonimage borders and the total number of pixels of the image divided by the positive number of pixels. For all quantification, a minimum of 4 images/tissue section/animal with 4 different animals per group were used. Data are means+/- SEM. Groups with no letter superscripts in common are significantly different from each other, P <0.05.
(XLSX)

S2 Table Carbonylated proteins identified in control and PTEN^LKO^ using LC-MS. (XLSX)

S3 Table Comparison of carbonylated proteins identified in PTEN^LKO^ livers to those identified in other murine models. (XLSX)

S4 Table Comparison of proteins identified in control and PTEN^LKO^ mice. (XLSX)

S5 Table Carbonylated proteins identified only in PTEN^LKO^. (XLSX)

## Abbreviations

4-HNE: 4-hydroxy-2-nonenal
4-ONE: 4-oxo-2-nonenal
Akt: Protein kinase B
ALT: alanine aminotransferase
CBR1/3: carbonyl reductase isoform 1 and 3
CID: collision-induced dissociation
CK: cytokeratin
CV: central vein
ETD: electron transfer dissociation
GST: glutathione S-transferase
HO-1: heme oxygenase
Keap1: Kelch-like ECH-associated protein 1
LE: whole cell extract
MDA: malondialdehyde
MMP: matrix metalloproteinases
MPO: myeloperoxidase
NAFLD: non-alcoholic fatty liver disease
NASH: nonalcoholic steatohepatitis
NQO1: NAD(P)H quinone dehydrogenase 1
Nrf2: nuclear factor erythroid-2-like-2
PT: portal triad
PTEN: phosphatase and tensin homolog deleted on chromosome 10
ROS: reactive oxygen species
