# Supplementary figures and images for: Elevated Nrf-2 responses are insufficient to mitigate protein carbonylation in hepatospecific PTEN deletion mice
Source: PLoS One. 2018 May 25;13(5):e0198139. doi: 10.1371/journal.pone.0198139 (PMC5969769; doi:10.1371/journal.pone.0198139)

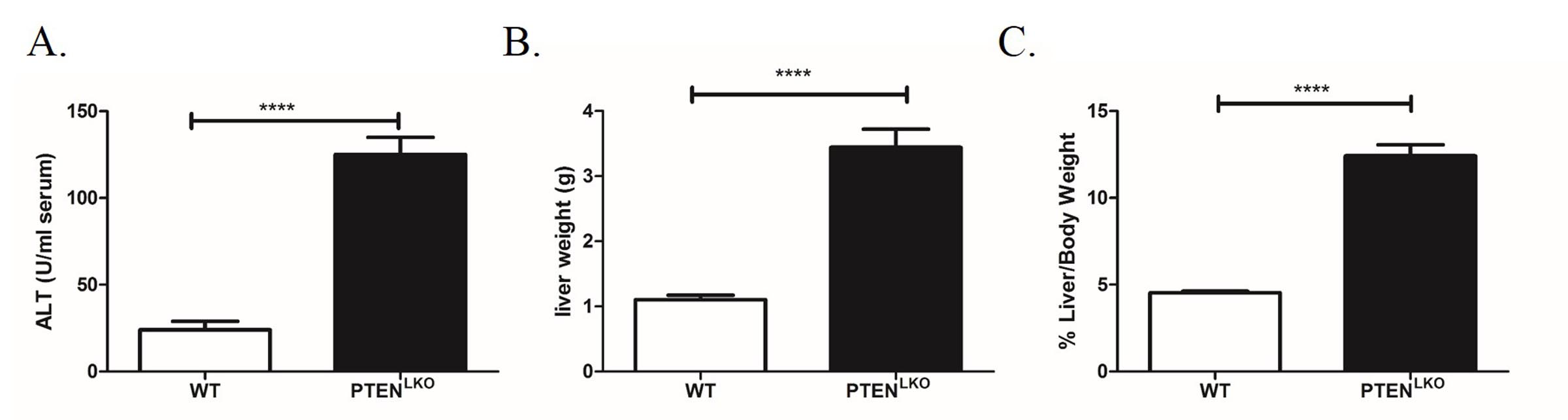

Supplement: S1 Fig — A. Alanine aminotransferase. B. Liver Weight. C. Liver to Body weight ratio. Data are means +/- SEM. N = 8 PTENLKO, 9 controls. (TIF) [file pone.0198139.s001.tif]
